# Effects of a Possible Pollinator Crisis on Food Crop Production in Brazil

**DOI:** 10.1371/journal.pone.0167292

**Published:** 2016-11-30

**Authors:** Samuel M. A. Novais, Cássio A. Nunes, Natália B. Santos, Ana R. D`Amico, G. Wilson Fernandes, Maurício Quesada, Rodrigo F. Braga, Ana Carolina O. Neves

**Affiliations:** 1 Departamento de Biologia Geral, Universidade Federal de Minas Gerais, Belo Horizonte, Minas Gerais, Brazil; 2 Laboratorio Nacional de Análisis y Síntesis Ecológica, Escuela Nacional de Estudios Superiores and Instituto de Investigaciones en Ecosistemas y Sustentabilidad, Universidad Nacional Autónoma de México, Morelia, Michoacán, México; 3 Departamento de Biologia, Universidade Federal de Lavras, Lavras, Minas Gerais, Brazil; 4 Instituto Chico Mendes de Conservação da Biodiversidade - ICMBio, Rondônia, Brazil; 5 Department of Biology, Stanford University, Stanford, United States of America; Embrapa, BRAZIL

## Abstract

Animal pollinators contribute to human food production and security thereby ensuring an important component of human well-being. The recent decline of these agents in Europe and North America has aroused the concern of a potential global pollinator crisis. In order to prioritize efforts for pollinator conservation, we evaluated the extent to which food production depends on animal pollinators in Brazil—one of the world’s agriculture leaders—by comparing cultivated area, produced volume and yield value of major food crops that are pollinator dependent with those that are pollinator non-dependent. In addition, we valued the ecosystem service of pollination based on the degree of pollinator dependence of each crop and the consequence of a decline in food production to the Brazilian Gross Domestic Product and Brazilian food security. A total of 68% of the 53 major food crops in Brazil depend to some degree on animals for pollination. Pollinator non-dependent crops produce a greater volume of food, mainly because of the high production of sugarcane, but the cultivated area and monetary value of pollinator dependent crops are higher (59% of total cultivated area and 68% of monetary value). The loss of pollination services for 29 of the major food crops would reduce production by 16.55–51 million tons, which would amount to 4.86–14.56 billion dollars/year, and reduce the agricultural contribution to the Brazilian GDP by 6.46%– 19.36%. These impacts would be largely absorbed by family farmers, which represent 74.4% of the agricultural labor force in Brazil. The main effects of a pollinator crisis in Brazil would be felt by the poorer and more rural classes due to their lower income and direct or exclusive dependence on this ecosystem service.

## Introduction

A great portion of the crops used for human consumption, such as fruits, vegetables, oilseeds, greens and grains, depend on wind, water, and animals for pollination. Seven out of the ten most important crops in the world, in terms of volume, are pollinated by wind (maize, rice and wheat) or have vegetative propagation (sugar cane, potato, beet, and cassava) [1]. However, it is estimated that 75% of the species grown for human consumption are pollinated by insect pollination [2]. The majority of these crops are fruits, which have experienced a continuous increase in production from 1961 to 2006 [3]. Furthermore, some species that are dependent on animal pollination are widely marketed, such as coffee and fruit of the Rosaceae family (apple, pear, plum, cherry, and almond) [4].

There is strong evidence of recent declines in wild and domesticated pollinators, as well as disruptions in the plant populations that rely upon them—which has been termed ‘the pollinator crisis’ [5]. Regional declines in pollinators have been recorded in the USA, where 59% of domesticated honeybee colonies were lost between 1947 and 2005 [6], and in central Europe, where the loss was about 25% between 1985 and 2005 [7]. In addition, the number of honeybee hives in the world has increased at a slower rate than the agricultural crops dependent upon pollinators (increases of ~45% and >300%, respectively), which implies a potential deficit of pollinators [8]. Evidence of the decline of wild pollinator communities is being recorded worldwide [9–12], including in Brazil [13, 14].

Multiple anthropogenic drivers, such as pesticides, introduced pathogens, climate change, and, primarily, land-use change have been implicated in insect-pollinator declines [15–17]. Land-use changes and agricultural intensification have major impacts on landscape structure, reducing diversity and the availability of pollinators due to increased habitat isolation, and reduction of floral resources and nesting areas in remnant habitats [11, 18]. In addition, the effects on pollinators are even greater when natural vegetation is replaced by monoculture plantations, which, when compared with more diverse systems, lack floral diversity and can limit the provisioning of resources required by pollinators throughout seasons [19, 20].

The occurrence of a pollinator crisis in Brazil is still uncertain but some studies have recently warned about its threats [13, 14]. Many processes currently operating in Brazil may pose a threat to the community of pollinators and the ecosystem service they provide, and this alone should be considered. Of broad importance are the processes related to land use change and habitat fragmentation. A recently approved revision in Brazil's Forest Code—the central piece of legislation regulating land use and management on private properties—predicts, for example, a 58% reduction in the need to restore illegally cleared forests (from 50 to 21 million hectares), and the permission to legally deforest over 88 million hectares [21]. The expansion of soy cultivation has become one of the main drivers of deforestation in the Cerrado and Amazon biomes, and it endangers biodiversity by fragmentation [22, 23], despite the reduction in deforestation in the Amazon Basin since 2004 [24, 25]. In terms of public lands, Brazil established itself as an environmental leader in the last few decades as it has produced the largest network of protected areas in the world [26]. However, these areas are being subjected to downsizing, reclassification and degazettement [27] in response to new pressures due to rising demands for hydropower and mineral resources [28]. A direct threat to pollinators is the use of agrotoxic compounds, such as glyphosate—a widely used herbicide in Brazilian agriculture—that reportedly causes behavioral changes in the honey bee, *Apis mellifera* [29]. Brazil ranks as the world leader in pesticide consumption, with glyphosate being used on 25 different crops [30]. Another potential threat is the introduction of exotic species, such as the European bee, *Bombus terrestris*, which was introduced into South America in 1970 to pollinate crops in Chile. From there, this bee spread to Argentina and Uruguay, and is likely to reach Brazil soon, causing unpredictable consequences as it may compete with native pollinators and loots the nectar of some flowers, hindering their reproduction and productivity [31].

If a pollinator crisis presents a gloomy outlook for the USA and the European Union (first and second largest food producers in the world, respectively), a similar scenario in Brazil would be equally troubling as it is considered the world’s fourth greatest food producer and third largest food exporter. Brazil is the world leader in coffee, sugar, and orange juice production and exports, and it leads in international sales of the soy complex (bran, oil and grain) [32]. The income generated by crops that depend in some way or another on animal pollinators is about US$ 45 billion, and the total contribution of pollinators corresponded to US$ 12 billion per year in Brazil [33]. In this study we evaluated the importance of pollinators to food production in Brazil by comparing pollinator dependent and pollinator non-dependent crops in terms of total planted area (ha), production weight (tons) and monetary value (US$), as well as average productivity (tons/ha) and the mean monetary value per area (US$/ha) of crops. We also determined the value of the ecosystem service of pollination based on the degree of pollinator dependence of crops and the estimated effects that a pollinator crisis would have in Brazil regarding potential decrease in agricultural production, economic losses, and impacts on Brazilian Gross Domestic Product and food security. Finally, we suggest some efforts for pollinator conservation in Brazil.

## Materials and Methods

We generated a list of current major cash crops in Brazil in terms of production volume using data from the website of the Brazilian Institute of Geography and Statistics (IBGE; http://www.sidra.ibge.gov.br/) for 2013, the most recent data available. Crops that benefit somehow from animal pollinators were considered *dependent*, and crops that do not benefit at all were considered *non-dependent* [2, 34]. To determine the importance of animal pollination to dependent crops we followed the classification used by [2, 34], which we complemented with updated information about pollinator dependency for Brazilian crops [33]. We classified crops into five pollinator dependency classes in accordance with the reduction in production caused by the total absence of pollinators (*Essential*, *High*, *Moderate*, *Small* and *Non-dependent*; Table 1). For example, in the *Essential* class, the absence of pollinators produces a reduction in production of ≥ 90% compared with the production of crops with pollinators present. Although coffee varieties vary in their dependence on pollinators, coffee was classified as *Moderate* since *Arabica* is the most commonly produced variety in Brazil in terms of volume and monetary value (S1 Appendix).

**Table 1:** Classification of crops according to their dependence on pollinators and production decrease in the total pollinator absence in two hypothetical scenarios of pollinator dependence of crops (optimistic and pessimistic).

Classes of crop dependence on pollinator	Total absence of pollinator	Optimistic scenario	Pessimistic scenario
Essential	≥ 90%	90%	100%
High	40% to < 90%	40%	89%
Moderate	10% to < 40%	10%	39%
Small	> 0 to < 10%	1%	9%
Non-dependent	0%	0%	0%

### Yield of pollinator dependent vs pollinator non-dependent crops

In order to identify more productive and/or more rentable crops, we compared total planted area (ha), production weight (tons), and monetary value (US$) between pollinator dependent and pollinator non-dependent crops. With regard to sugarcane, we considered only the proportion of the 2013/2014 harvest used for sugar production in the analyses (which was 49%, the rest was used for ethanol production) [35]. Also, we calculated the average productivity (tons/ha) and the mean monetary value per area (US$/ha) for pollinator dependent and non-dependent crops and performed Generalized Linear Models (GLM) on R Program [36] to evaluate if there were differences between these yields. Species with unknown pollinator dependency were not considered in this analysis.

### Valuation of pollination service and its loss in hypothetical scenarios

To evaluate the economic losses under different hypotheses of decline of pollinator populations in Brazil, we performed a simulation based on crop dependence on pollinators [2]. We calculated the proportion of the production and the monetary value of the crops that depend on pollinators [37] in two hypothetical scenarios—one optimistic and one pessimistic—as shown in Table 1. For example, a crop with an *Essential* dependency level (≥90%) would reduce the production/value by 90% in an optimistic scenario and by 100% in a pessimistic scenario. A crop with a *Moderate* dependency level (10% to <40%) would reduce the production/value by 10% in an optimistic scenario and by 39% in a pessimistic scenario. We discussed the impact of economic losses on the contribution of agriculture to GDP (Gross Domestic Product) of the year 2013 [38] and on main exported agricultural commodities [32].

### Brazilian’s food security

To evaluate the influence of crop pollinator dependence on food security in Brazil, we used data on a *monthly per capita* consumption of several food items per family income class [39].

## Results

### Crop species

**Fig 1:**
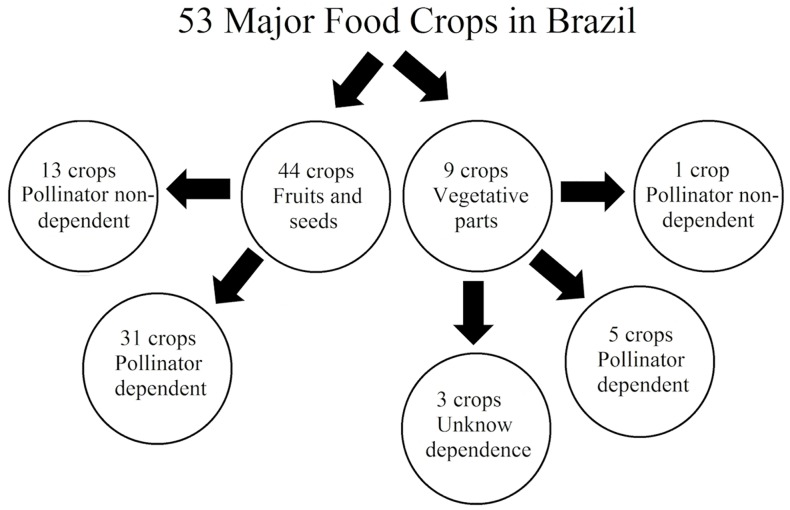
Summary of pollinator dependence of the 53 most produced food crops in Brazil. Crops that produce food from fruits/seeds or vegetative parts and benefit somehow from animal pollinators were considered *dependent*, and crops that do not benefit at all were considered *non-dependent*.

A total of 53 food types were identified as the major food crops in Brazil (S1 Appendix). Among these, 44 produce fruits or seeds, of which 31 benefit from pollinators. Nine species produce food from their vegetative parts, yet five of these were still benefited by pollinators (Fig 1). Out of 36 crops that are benefited by pollinators, 29 were classified with regard to their pollinator dependence level, with 32.5% (14) being classified as *Essential* and *High*, 19% (8) as *Moderate*, 16% (7) as *Small* and 32.5% (14) as *Non-dependent* (S1 Appendix).

### Yield of pollinator dependent vs pollinator non-dependent crops

Pollinator non-dependent crops are responsible for 75% of the total production (t) of the major food crops in Brazil (Table 2), with sugarcane alone being responsible for 58.87% of the total food volume produced. However, cultivated area and monetary value (US$) of pollinator dependent crops are larger than non-dependent crops (Table 2). Soybean alone, classified as of *Moderate* pollinator dependence, corresponds to 42.81% of the total cultivated area. We found no statistical difference in the average productivity between the two groups (Table 2, p = 0.25, F = 0.35, D.F. = 48). The mean monetary value per area of pollinator dependent crops is three times greater than that of pollinator non-dependent crops (Table 2, p = 0.021, F = 5.68, D.F. = 48). Models (GLMs) were adjusted with Gaussian error distribution.

**Table 2:** Absolute and relative values of pollinator dependent and pollinator non-dependent crops in Brazil.

	Pollinator dependent crops (36 species)	Pollinator non-dependent crops (14 species)	Total
Area (ha) 10^6^	38.62 (59.16%)	26.67 (40.84%)	65.29
Production (t) 10^6^	158.38 (24.77%)	480.96 (75.23%)	639.34
Value (US$) 10^9^	42.56 (67.99%)	20.04(32.01%)	62.60
Production (t)/Area (ha) (SD)	15.36 (±14.37)	12.30 (±20.09)	-
Value (US$)/Area (ha) (SD)	5.07 (±5.52)*	1.41 (±1.65)	-

Production/area and value/area = mean and standard deviation (SD).

* Represents significant differences (p < 0.05).

### Valuation of pollination ecosystem service and its loss

In the hypothetical scenario of a pollinator crisis in Brazil, the country would have an estimated reduction in food production of 16.55–51 million tons in the optimistic and pessimistic scenarios, respectively, which means a decline from 13.5%– 41.59% in production of food derived from pollinator dependent crops and from 2.59%– 7.97% of total production of major crops (Table 3). Impacts would be even more significant in monetary terms, as Brazil’s revenue would be reduced US$ 4.86–14.56 billion per year, which means a decline of 13.84%– 41.46% in the income of pollinator dependent crops and 7.76%– 23.25% of total income of major food crops in the country. Based on these data, Brazil would have a reduction of, at least, 6.46%– 19.36% in agricultural contribution to GDP.

**Table 3:** Losses in production for 29* major pollinator dependent food crops in Brazil, under optimistic and pessimistic hypothetical scenarios of pollinator loss.

	Pollinator dependent crops	Optimistic scenario	Pessimistic scenario
Area (ha) 10^6^	36.27	36.27	36.27
Production (t) 10^6^	122.65	106.09	71.64
Value (US$) 10^9^	35.11	30.25	20.55

*Annatto, Apple, Avocado, Bean, Broad bean, Cashew nut, Cocoa, Coconut, Coffee, Cowpeas, Fig, Groundnut, Guarana, Guava, Lemon, Melon, Oil palm, Orange, Papaya, Passion fruit, Peach, Pear, Persimmon, Quince, Soybean, Sunflower, Tangerine, Tomato, Watermelon.

### Pollinator dependence on food security

Sixty percent of the food consumed by Brazilians (in grams *per capita*) derive from pollinator dependent crops, regardless of family income class (Table 4), and represents the sum of products derived from 21 of the 53 most produced crops in Brazil (Apple, Banana, Bean, Cassava, Cocoa, Coffee, Grape, Maize, Mango, Orange, Papaya, Pineapple, Potato, Rice, Soybean, Sugar cane, Sweet potato, Tangerine, Tomato, Watermelon, Wheat; S1 Appendix).

**Table 4:** Brazilian *monthly per capita* consumption of products derived from pollinator dependent and non-dependent crops, according to household income class.

Product	Up to US$ 120	Between US$ 120 and US$ 233	Between US$ 233 and US$ 444	More than US$ 444	Total Mean
Pollinator dependent	60.11%	60.12%	60.67%	58.87%	59.98%
Pollinator non-dependent	39.89%	39.88%	39.33%	41.13%	40.02%

## Discussion

Our study indicates that 68% of the major food crops of Brazil are dependent on pollinators. The percentage of pollinator dependent crops in this study is greater than the value found in another study for Brazil (~60%)[33], mainly because they included plants of other economic importance in their data set, such as crops used for clothing, livestock and biofuel. Our value is lower than that found for the European Union (84%), Mexico (80%) and the world average (74%) [2, 34, 40], but it is certainly an underestimate since the less economically important food crops in Brazil were not included in the analysis due to lack of updated official information regarding area, monetary value and production. For example, of the 75 food species dependent on insect pollination of Brazil [41], only 19 species contain specific information of the area, monetary value and production and that is why some other indigenous crops, such as the açai tree (*Euterpe oleraceae*), the Brazil nut tree (*Bertolettia excelsa*) and the cupuaçu (*Theobroma grandiflorum*), were unfortunately excluded from our study.

The method for determining production value used in our study is proposed as an alternative to the attributable net income (ANI) method [42] when information on costs of different production factors, such as fertilizers or pesticides, is not available, which is especially true for studies that deal with a wide variety of crops [34, 43–45]. We acknowledge the fact that the ANI method might reflect more accurately the value of pollination service because it subtracts the cost of inputs to crop production from the value of pollination, but most of the studies surveyed did not have information on the cost of inputs for crop production.

In a similar study conducted for Mexico, a highly populated country like Brazil where the livelihoods of people strongly rely on the provisioning of pollinator-dependent food, we could expect to find similar patterns for the evaluated metrics between dependent and non-dependent pollinator crops. For Brazil, pollinator dependent crops are responsible for only one quarter of the country’s total production (in terms of tons of the major food crops), although such crops occupy a larger area and have greater mean monetary value per area than non-dependent crops. In fact, in Brazil, pollinated crops represent a smaller proportion of total production than in Mexico (38%) and the world as a whole (35%) [2, 34]. We also could expect to find greater productivity of pollinated crops in comparison to non-dependent crops based on the data from Mexico, where the productivity of the former was double of the latter [34]. However, we found no statistically significant difference between pollinator dependent and non-dependent crops with regard to productivity. These results are primarily determined by the sugarcane cultivation, which represents more than half of the food volume produced in Brazil, and whose yield is six times higher than the average for non-dependent pollinator crops.

The area occupied by pollinator dependent crops in Brazil corresponds to 59.15% of the total cultivated area, mainly because of soybean. In the last decade, the increased demand in the international market, especially by China, for soybeans, has driven an increase in production and the development of new varieties that have allowed the expansion of soybean crops to areas of Cerrado and Amazon [46]. Brazilian production, which was 59 million tons in 2008, increased to 81 million in 2013, occupying ca. 28 million hectares—an area larger than the sum of the other four major grains produced in the country, i.e. rice, beans, corn and wheat [47].

The mean monetary value per area of pollinator dependent crops in Brazil is almost three-times that of non-dependent crops, with 67.99% of the resources generated by the major crops in Brazil coming from them (i.e. about US$ 42 billion in 2013). As discussed previously, this value is certainly an underestimate due to the inexistence of data for at least 58 out of 75 minor pollinator dependent Brazilian crops [41]. In Mexico, dependent crops represent 54% of the monetary value generated by agriculture [40], while 39% of the world production value comes from dependent crops [37]. The higher percentage of monetary value generated by dependent crops in Brazil could be due to the strong contribution of soybean and coffee production, both pollinator dependent crops. Considering world production, a greater percentage of resources is derived from cereals [37], which are mostly non-dependent crops; while the extensive corn production, which is wind-pollinated, has an important contribution to the percentage of resources generated by non-dependent crops in Mexico [34]. These different agriculture profiles could explain the lower percentage of resources generated by pollinator dependent crops in the world as a whole, as well as in individual countries such as Mexico, in comparison to Brazil.

We highlight the impact of a hypothetical crisis on two pollinator dependent crops that are highly relevant Brazilian exports: soybean (*Moderate*) and coffee (*Moderate—Coffea arabica*, main exported coffee). In 2013, products of the soybean complex and coffee totaled US$ 30.96 billion and US$ 5.28 billion in exports, respectively, representing together 41.83% of total agricultural exports [32]. Based on the scenarios proposed in this study, and a possible decrease in production of these two crops, we assume a proportional reduction in the value for export, which would be between US$ 3.1 and US$ 12.07 billion for soybean and between US$ 528 million and US$ 2.06 billion for coffee in the optimistic and pessimistic scenarios, respectively. These values represent a decrease of 4.18–16.31% and a reduction of US$ 3.62–14.13 billion of agricultural export revenue. Furthermore, these two crops maintain a lot of employees in Brazil’s agricultural system, with the coffee sector, for example, employing at least eight million people [48].

Brazil is the major producer (30% of world’s production) and exporter of coffee in the world [49]. In a pessimist scenario of a possible pollinator crisis in Brazil, the production of coffee would have decreased 40%, which means that the world would have 12% less coffee to consume. Certainly, this event would impact global prices and the economies of importers and other exporters of coffee. Some importers that re-export coffee (like Germany, the second greatest importer of coffee in the world) would have a more significant impact on their economies, as they depend on the original producers to generate income from coffee [49]. As with coffee, Brazil is also the major producer and exporter of soybean [49], and we can imagine similar global consequences to prices and economies due to a possible pollinator crisis in Brazil.

Assumptions about the responses of consumers and farmers to declines in pollination services are highly speculative. The decline in pollinator supply may occur at different spatial or temporal scales, and it is unclear how consumers would respond to a changing supply of different pollinator-dependent crops independently of one another [42, 43, 50]. For Brazil, in general, we believe that the impacts of pollinator-dependent production losses will be largely absorbed by family farmers, considering that family agriculture employs 74.4% of the total agricultural workforce and accounts for a large portion of agricultural production [51]. For example, family farmers account for 87% of cassava, 70% of beans, and 38% of coffee produced in Brazil [51]. These foods are benefited by animal pollination and are among the crop foods most consumed by the Brazilian population. However, as possible alternatives, farmers can switch to less pollinator dependent crops or, as an immediate response, adjust the market price to outweigh the production losses [42]. These alternatives will mostly affect consumers of crops, while other affected producers could lose or gain depending on the magnitude of the price effect. [42].

There was no difference between the proportion of pollinator dependent and non-dependent agricultural products consumed by different income classes in Brazil. However, the effects of a possible reduction in food production and the consequent price increase would be mainly absorbed by the lower income and rural classes, because they are more economically vulnerable and depend directly or exclusively on the environmental service of pollination [52]. Similarly, a previous study also showed that the poor and rural people of Mexico would face immediate food security risks under a pollinator crisis as they directly rely on pollinator-dependent agricultural products [34]. Among the ten food crops most consumed by the urban and rural populations in Brazil, six are dependent to some degree on pollinators. Regarding the lowest income class (*monthly per capita* income of up to US$ 120), seven products most consumed depend in some way on pollinators [39]. Finally, whereas the World Health Organization (WHO) recommends a daily intake of 400g/person of fruits, vegetables and greens for a healthy, nutritional diet [53], and most of these crops depend on pollinators, a possible reduction of these agents would threaten the nutritional security of Brazilians.

Our analysis makes clear the necessity of adopting public policies and management measures to prevent a potential pollinator crisis in Brazil. These actions should be effective for the long term, rather than short-sighted measures such as increased planting of the major crops affected with decreased production [54]. Such short-sighted alternatives lead to greater habitat loss and fragmentation of natural environments, which contributes to further pollinator loss in the long run. Based on the existing literature and the experience of other countries, some recommendations are presented below.

Preservation of remaining natural and semi-natural habitats forming heterogeneous agricultural landscapes favors pollination [55–57], since pollination decreases with increasing distance between cultivation and fragments of natural habitat, especially for intensive monoculture systems [58, 59]. The presence of areas of native vegetation maintains pollinators close to cultivated areas, since they serve as an additional source of nectar and pollen through flowering during times when crops are not, and provide areas for resting, nesting and reproduction of pollinators [60]. In this context, the management of cultivated areas in conjunction with natural areas, or agroforestry systems, can be a good practice for avoiding pollinator reduction.

Unfortunately, Brazil has adopted policies in opposite direction. The recently revised Brazilian Forest Code, for example, allows for the reduction of declared protected areas within private properties [61]. Moreover, the slowdown in the creation of protected areas also threatens the maintenance of pollinators at the landscape level, especially in the Cerrado biome of which only 7% is protected [21] and where the main export crops (coffee and soybeans) are increasingly expanding [23].

Based on the success of the moratorium on soy cultivation in newly deforested areas in the Amazon [23, 62], international sanctions, including requirements to adopt practices beneficial to pollinators by farmers, especially those who work with large-scale farming, can contribute to the conservation of pollinators in Brazil. Stricter rules for the release of financing, including conditions such as the maintenance of the quality of the environment and the use of less aggressive practices, as well as economic benefits, such as payment for environmental services to producers, have great potential for generating positive changes [63, 64].

Other damages that can be mitigated include those caused by pesticides, which occur in many different ways [65, 66]. When used improperly, agrochemicals have extremely negative impacts on pollinators, including their diversity and abundance and thus their pollination efficiency [67]. Therefore, as an alternative, and to help prevent a pollinator crisis in Brazil, more research is needed into favoring agroecological systems and organic production, in addition to considering a decrease in the use of these chemicals. The development of pesticides that take into account the potential side effects to pollinators is also essential for Brazil to remain among the world's largest agricultural producers.

Finally, it is essential that continuous monitoring be conducted with standardized sampling in order to verify long-term trends in the population dynamics of pollinators, as has been proposed by organizations like International Pollinators Initiative [4, 15]. According to the precautionary principle, management actions must be taken to prevent this hypothetical crisis from becoming a reality.

We conclude that Brazil is vulnerable to a pollinator crisis, as its economy is deeply based on agriculture, and its production largely relies on pollinators. More than half of the cultivated area and total yield of major Brazilian food crops, as well as ~70% of the revenue generated by the major crops, come from cultivars that exhibit some degree of pollinator dependence. This revenue, as well as the mean monetary value per area of pollinator dependent crops in Brazil, is much larger than the world average and also larger than that of other individual countries such as Mexico. In addition, economic harms of a possible pollination service loss were estimated in the billions of US$ per year for 29 of the major pollinator dependent crops in Brazil. The consequences resulting from a decrease of Brazilian agricultural production will affect family farmers to large producers and affect all sectors of society, particularly the poor and the rural population.

## Supporting Information

S1 Appendix Detailed description of the 53 major food crops produced in Brazil in 2013. (DOCX)
