# Correction: Effects of a Possible Pollinator Crisis on Food Crop Production in Brazil

**DOI:** 10.1371/journal.pone.0197396

**Published:** 2018-05-09

**Authors:** Samuel M. A. Novais, Cássio A. Nunes, Natália B. Santos, Ana R. D`Amico, G. Wilson Fernandes, Maurício Quesada, Rodrigo F. Braga, Ana Carolina O. Neves

There are errors in Table 2. The values in line 4 “Production (t)/Area (ha) (SD)” and line 5 “Value (US$)/Area (ha) (SD)” are incorrect. Please see the corrected Table 2 here.

**Table 2:** Absolute and relative values of pollinator dependent and pollinator non-dependent crops in Brazil.

	Pollinator dependent crops (36 species)	Pollinator non-dependent crops (14 species)	Total
Area (ha) 10^6^	38.62 (59.16%)	26.67 (40.84%)	65.29
Production (t) 10^6^	158.38 (24.77%)	480.96 (75.23%)	639.34
Value (US$) 10^9^	42.56 (67.99%)	20.04 (32.01%)	62.60
Production (t)/Area (ha) (SD)	15.32 (±14.41)	12.30 (±20.09)	-
Value (US$)/Area (ha) (SD)	5021 (±5536)*	1412 (±1654)	-

Production/area and value/area = mean and standard deviation (SD).

* Represents significant differences (p < 0.05).
