# Supplementary material for: Effects of a Possible Pollinator Crisis on Food Crop Production in Brazil
Source: PLoS One. 2016 Nov 30;11(11):e0167292. doi: 10.1371/journal.pone.0167292 (PMC5130262; doi:10.1371/journal.pone.0167292)
Supplement: S1 Appendix — (DOCX) [file pone.0167292.s001.docx]

S1 APPENDIX. Detailed description of the 53 major food crops produced in Brazil in 2013.

S1 TABLE. Relative values of production situation, consumed part, pollination effect and pollinator dependence degree for the 53 major pollinator dependent and non-pollinator dependent food crops in Brazil in 2013.

| **Food Crop (English/Portuguese)** | **Scientific name** | **Consumed Part** | **Pollination effect [2, 33]** | **Production Volume (t)** | **Production Value (1000 US$)** | **Planted Area (ha)** | **Production (t)/Area (ha)** | **Value (1000 US$)/Area (ha)** |
| --- | --- | --- | --- | --- | --- | --- | --- | --- |
|  |  |  |  |  |  |  |  |  |
| Annatto/Urucum | *Bixa orellana* (L.) | Seed | Dependent - Essential | 39545 | 3772.12 | 10530 | 3.76 | 0.36 |
| Apple/Maça* | *Pyrus malus* (L.) | Fruit | Dependent - High | 1231472 | 324865.06 | 38292 | 32.16 | 8.48 |
| Avocado/Abacate | *Persea americana* (Mill.) | Fruit | Dependent - High | 157482 | 48169.87 | 9707 | 16.22 | 4.96 |
| Banana* | *Musa sapientum* (L.), *M. cavendishii* (Lamb. exPaxton), *M. nana* (Lour.), *M. paradisiaca* (L.) | Fruit | Dependent - Unknown | 6892622 | 1639174.04 | 490628 | 14.05 | 3.34 |
| Barley/Cevada | *Hordeum vulgare*(L.) | Seed | Non-dependent | 330682 | 61187.82 | 88406 | 3.74 | 0.69 |
| Bean/Feijão* | *Phaseolus lunatus* (L.), *P.angularis* (Willd.) *W. Wight, P.aureus* (Wall.), *P. mungo* (L.), *P. coccineus* (L.), *P. calcaratus* (Roxb.), *P. aconitifolius* (Jacq.), *P. acutifolius* (A. Gray) | Seed | Dependent - Small | 2892599 | 2226152.24 | 3041299 | 0.95 | 0.73 |
| Broad bean/Fava | *Vicia faba* (L.) | Seed | Dependent - Moderate | 7957 | 15831.41 | 25542 | 0.31 | 0.62 |
| Cashewnut/Castanha de caju | *Anacardium occidentale* (L.) | Fruit | Dependent - Moderate | 109679 | 51376.28 | 708808 | 0.15 | 0.07 |
| Cassava/Mandioca* | *Manihot esculenta* (Crantz), *M. palmata* (Mull. Arg.) | Vegetative | Dependent - Unknown | 21484218 | 3246958.97 | 1560263 | 13.77 | 2.08 |
| Cocoa/Cacau* | *Theobroma cacao* (L.) | Fruit | Dependent - Essential | 256186 | 389114.74 | 692435 | 0.37 | 0.56 |
| Coconut/Coco da baía | *Cocos nucifera* (L.) | Fruit | Dependent - Moderate | 1926857 | 331725.96 | 259015 | 7.44 | 1.28 |
| Coffee/Café* | *Coffea arabica* (L.) | Fruit | Dependent - Moderate | 2320343 | 3325964.42 | 1621768 | 1.43 | 2.05 |
| Coffee/Café* | *C. canephora* (P.) | Fruit | Dependent - High | 644195 | 783116.03 | 472489 | 1.36 | 1.66 |
| Cowpeas/Ervilha | *Vigna unguiculata* ((L.) Walp.) | Seed | Dependent - Small | 2992 | 1888.78 | 1259 | 2.38 | 1.50 |
| Fig/Figo | *Ficuscarica* (L.) | Fruit | Dependent - Moderate | 28253 | 20649.68 | 2814 | 10.04 | 7.34 |
| Garlic/Alho | *Allium sativum* (L.) | Vegetative | Dependent - Unknown | 102232 | 183890.38 | 9567 | 10.69 | 19.22 |
| Grape/Uva* | *Vitis spp.* (L.) | Fruit | Non-dependent | 2120893 | 461389.42 | 79759 | 26.59 | 5.78 |
| Groundnut/Amendoim | *Arachis hypogaea* (L.) | Seed | Dependent - Small | 389783 | 153787.50 | 121102 | 3.22 | 1.27 |
| Guarana/Guaraná | *Paullinia cupana* (Kunth) var. sorbilis | Fruit | Dependent - High | 3662 | 10427.24 | 13916 | 0.26 | 0.75 |
| Guava/Goiaba | *Psidium guajava*(L.) | Fruit | Dependent - High | 349615 | 125840.38 | 15034 | 23.25 | 8.37 |
| Heart of palm/Palmito | *Euterpe edulis* Mart. | Vegetative | Unknown | 106418 | 99320.51 | 17331 | 6.14 | 5.73 |
| Lemon/Limão | *Citrus latifolia* Tanaka | Fruit | Dependent - Small | 1169370 | 220049.36 | 46036 | 25.40 | 4.78 |
| Maize/Milho* | *Zea mays* (L.) | Seed | Non-dependent | 80273172 | 8565095.19 | 15708367 | 5.11 | 0.55 |
| Mango/Manga* | *Mangifera indica* (L.) | Fruit | Non-dependent | 1163000 | 290681.73 | 70718 | 16.45 | 4.11 |
| Melon/Melão | *Cucumis melo* (L.) | Fruit | Dependent - Essential | 565900 | 160785.90 | 22062 | 25.65 | 7.29 |
| Oat/Aveia | *Avena sativa* (L.) | Seed | Non-dependent | 520397 | 72791.35 | 243181 | 2.14 | 0.30 |
| Oil palm/Dendê | *Elaeis guineensis* (L.) | Fruit | Dependent - Small | 1246835 | 100589.74 | 108638 | 11.48 | 0.93 |
| Olive/Azeitona | *Olea europaea* (L.) | Fruit | Non-dependent | 265 | 191.99 | 146 | 1.82 | 1.31 |
| Onion/Cebola | *Allium cepa* (L.), *A. ascalonicum* (L.), *A. fistulosum* (L.) | Vegetative | Dependent - Unknown | 1538929 | 418281.41 | 57587 | 26.72 | 7.26 |
| Orange/Laranja* | *Citrus aurantium* (L.), *C. sinensis* (Citrus) | Fruit | Dependent - Moderate | 17549536 | 1527443.59 | 719360 | 24.40 | 2.12 |
| Papaya/Mamão* | *Carica papaya* (L.) | Fruit | Dependent - Small | 1582638 | 387749.04 | 32139 | 49.24 | 12.06 |
| Passion fruit/Maracujá | *Passiflora edulis* (Sims) | Fruit | Dependent - Essential | 838244 | 298725.64 | 58089 | 14.43 | 5.14 |
| Peach/Pessego | *Prunus persica* ((L.) Stokes) | Fruit | Dependent - High | 312059 | 69777.56 | 18092 | 17.25 | 3.86 |
| Pear/Pera | *Pyrus communis* (L.) | Fruit | Dependent - High | 30312 | 7076.28 | 1680 | 18.04 | 4.21 |
| Persimmon/Caqui | *Cavanillea philippensis* (Desr.), *Diospyros kaki* (Diospyros kaki) | Fruit | Dependent - Moderate | 173169 | 72267.95 | 8554 | 20.24 | 8.45 |
| Pineapple/Abacaxi* | *Ananas comosus* ((L.) Merr.) | Fruit | Dependent - Unknown | 1655887 | 594329.81 | 64421 | 25.70 | 9.23 |
| Potato/Batata-inglesa* | *Solanum tuberosum* (L.) | Vegetative | Dependent - Unknown | 3553772 | 1235793.27 | 128118 | 27.74 | 9.65 |
| Quince/Marmelo | *Cydonia oblonga* (Mill.), *C. japonica* ((Thunb.) Pers.) | Fruit | Dependent - High | 633 | 244.55 | 126 | 5.02 | 1.94 |
| Rice/Arroz* | *Oryza spp.* (L.) | Seed | Non-dependent | 11782549 | 2418279.81 | 2386821 | 4.94 | 1.01 |
| Rye/Centeio | *Secale cereale* (L.) | Seed | Non-dependent | 5743 | 1060.26 | 3844 | 1.49 | 0.28 |
| Sorghum/Sorgo | *Sorghum bicolor* (L.) Moench.) | Seed | Non-dependent | 2126179 | 171729.49 | 802020 | 2.65 | 0.21 |
| Soybean/Soja* | *Glycine max* ((L.) Merr.), *G. soja* (Siebold&Zucc.) | Seed | Dependent - Moderate | 81724477 | 22094347.12 | 27948605 | 2.92 | 0.79 |
| Sugar cane/Cana de açúcar* | *Saccharum spp*. (L.) | Vegetative | Non-dependent | 768090444 | 13764939.10 | 10223043 | 75.13 | 1.35 |
| Sunflower/Girassol | *Helianthus annuus* (L.) | Seed | Dependent - High | 108838 | 29947.44 | 69330 | 1.57 | 0.43 |
| Sweet potato/Batatadoce* | *Ipomoea batatas* ((L.) Lam.) | Vegetative | Dependent - Unknown | 505350 | 132338.46 | 39393 | 12.83 | 3.36 |
| Tangerine/Tangerina* | *Citrus reticulata* (Blanco) | Fruit | Dependent - Small | 636089 | 300583.01 | 50882 | 12.50 | 5.91 |
| Tea/Chá da índia | *Camellia sinensis* ((L.) Kuntze) | Vegetative | Unknown | 3282 | 797.76 | 372 | 8.82 | 2.14 |
| Tomato/Tomate* | *Lycopersicon esculentum* (Mill.) | Fruit | Dependent - High | 4187646 | 1673238.46 | 62782 | 66.70 | 26.65 |
| Triticale/Triticale | *Triticosecale rimpaui* (C. Yen& J.L. Yang) | Seed | Non-dependent | 62531 | 8500.64 | 27093 | 2.31 | 0.31 |
| Walnut/Noz | *Carya illinoinensis* ((Wangenh.) K. Koch) | Fruit | Non-dependent | 5228 | 7368.59 | 2844 | 1.84 | 2.59 |
| Watermelon/Melancia* | *Citrullus lanatus* (Citrullus) | Fruit | Dependent - Essential | 2163501 | 355124.04 | 93190 | 23.22 | 3.81 |
| Wheat/Trigo* | *Triticum spp.* (L.) | Seed | Non-dependent | 5738473 | 1220930.77 | 2225401 | 2.58 | 0.55 |
| White pepper/Pimenta do reino | *Piper nigrum* (L.) | Seed | Non-dependent | 469264 | 13561.54 | 18474 | 25.40 | 0.73 |
| Yerba mate/Erva mate | *Ilex paraguariensis* (A. St.-Hil.) | Vegetative | Unknown | 515451 | 130294.23 | 74421 | 6.93 | 1.75 |
| Sugar cane/Cana de açúcar** | *Saccharum spp*. (L.) | Vegetative | Non-dependent | 376364317.6 | 6744820.16 | 5009291.07 | 75.13 | 1.35 |

*Species mentioned in IBGE survey about *monthly per capita* consumption of various foods (IBGE, 2011).

**Considering only sugar cane production regarding to food production (49% of produced volume).

IBGE(http://www.sidra.ibge.gov.br/)
